# HLA-DR expression on monocytes and systemic inflammation in patients with ruptured abdominal aortic aneurysms

**DOI:** 10.1186/cc5017

**Published:** 2006-08-09

**Authors:** Jan Willem Haveman, Aad P van den Berg, Eric LG Verhoeven, Maarten WN Nijsten, Jan JAM van den Dungen, T Hauw The, Jan Harm Zwaveling

**Affiliations:** 1 Department of Surgery; Surgical Intensive Care Unit, University Medical Center Groningen, University of Groningen, Hanzeplein, 9700 RB Groningen, The Netherlands; 2 Department of Gastoenterology and Hepatology, University Medical Center Groningen, University of Groningen, Hanzeplein, 9700 RB Groningen, The Netherlands; 3 Department of Surgery; Vascular Surgery, University Medical Center Groningen, University of Groningen, Hanzeplein, 9700 RB Groningen, The Netherlands; 4 Department of Clinical Immunology, University Medical Center Groningen, University of Groningen, Hanzeplein, 9700 RB Groningen, The Netherlands

## Abstract

**Introduction:**

Mortality from ruptured abdominal aortic aneurysms (RAAA) remains high. Severe systemic inflammation, leading to multi-organ failure, often occurs in these patients. In this study we describe the level of HLA-DR expression in a consecutive group of patients following surgery for RAAA and compare results between survivors and non-survivors. A similar comparison is made for IL-6 and IL-10 levels and Sequential Organ Failure Assessment (SOFA) scores.

**Methods:**

This is a prospective observational study. Patients with RAAA were prospectively analysed. Blood samples were collected on days 1, 3, 5, 7, 10 and 14. The fraction of CD-14 positive monocytes expressing HLA-DR was measured by flow-cytometry. IL-6 and IL-10 levels were measured by ELISA.

**Results:**

The study included 30 patients with a median age of 70 years, of which 27 (90%) were men. Six patients died from multiple organ failure, all other patients survived. The SOFA scores were significantly higher in non-survivors on days 1 through 14. HLA-DR expression on monocytes was significantly lower on days 3, 5, 7, 10 and 14 in non-survivors. IL-6 and IL-10 levels were significantly higher in non-survivors on day 1 and days 1 and 3, respectively.

**Conclusion:**

HLA-DR expression on monocytes was decreased, especially in non-survivors. All patients with RAAA displayed a severe inflammatory and anti-inflammatory response with an increased production of IL-6 and IL-10. Poor outcome is associated with high levels of IL-6 and IL-10 and a high SOFA score in the first three days after surgery, while low levels of HLA-DR expression are observed from day three after RAAA repair.

## Introduction

Mortality in patients following repair of a ruptured abdominal aortic aneurysm (RAAA) remains high (30% to 70%), despite important advances in emergency medicine, anaesthesiology, surgery and intensive care [1-5]. The postoperative course of patients after RAAA repair is almost always characterized by systemic inflammation, sometimes leading to multiple-organ failure, a prolonged intensive care unit (ICU) stay and a high mortality [6-8]. Down-regulation of HLA-DR expression on monocytes has been reported in different groups of surgical patients and has been associated with septic complications and increased mortality [9-13]. We studied the expression of HLA-DR on monocytes in patients following surgery for RAAA, taking into account levels of IL-6 and IL-10 and Sequential Organ Failure Assessment (SOFA) scores. The primary aim of this study was to describe the level of HLA-DR expression in these patients and to establish, if possible, whether low HLA-DR expression was associated with increased mortality as a result of secondary infections.

## Materials and methods

### Patients and design of the study

Patients with RAAA who survived surgery were prospectively analysed and included in the study. Patients who underwent endovascular treatment were excluded. Cases were only classified as RAAA when an aortic aneurysm and retroperitoneal or intraperitoneal blood were present. The study was approved by our Medical Ethics Committee. Written informed consent was obtained from a family member.

For each patient, one healthy employee of the laboratory served as normal control.

On days 1, 3, 5, 7, 10 and 14, 10 ml of EDTA blood was withdrawn from the patient and HLA-DR expression on monocytes was analysed immediately. For IL-6 and IL-10 measurements blood was kept on ice, centrifuged at 1,655 *g *at 4°C for 10 minutes and stored at -80°C until analysis.

Acute Physiology and Chronic Health Evaluation (APACHE)-II scores were calculated on ICU admission [14]. The SOFA score was measured daily after surgery [15]. ICU-acquired infections were defined according to the criteria issued by the Centres for Disease Control and Prevention. All infections were recorded prospectively. Sepsis was defined according to Bone and colleagues [16].

### Laboratory analysis

C-reactive protein (normal value <5 mg/dl) and white blood cell count (normal value 4 to 10 × 10^9^/l) were measured every day.

IL-6 and IL-10 were measured by ELISA in 26 patients (21 survivors and five non-survivors), using a monoclonal antibody against human IL-6 (Sanquin, Amsterdam, the Netherlands) or IL-10 (BD Pharmingen, Alphen a/d Rijn, the Netherlands).

The percentage of CD-14 positive monocytes expressing HLA-DR was measured by flow-cytometry. Monoclonal antibodies against CD-14 antigen (anti-CD-14-PE, Immuno Quality Products, Groningen, the Netherlands) were used to set a gate for monocytes. The percentage of HLA-DR on monocytes was determined using anti-HLA-DR fluorescein isothiocyante (Becton Dickinson Immunocytometry Systems, San Jose, CA, USA), with an IgG2a isotype control (IgG2a FITC, Immuno Quality Products). A live gate was set using forward and sideward scatter characteristics. A monocyte gate was set by the CD-14+ group. Data were analysed using Cell Quest software (Becton-Dickinson).

### Statistics

Data are given as median with interquartile range. Differences between categorical variables were tested with Chi-square analysis. The Mann-Whitney *U *or Kruskal-Wallis test was performed to calculate differences in continuous variables. For detection of correlation we used Spearman's rank correlation test. The rank correlation coefficients were averaged after z-transformation. *P *values < 0.05 were regarded as statistically significant.

## Results

### Patients

During the course of the study 46 patients with RAAA were admitted to our Hospital. All patients were operated upon. Sixteen patients were not included in this study: six were endovascular treated, five died during surgery, four patients were not included because of absence of one of the primary investigators (JWH or APvdB), and for one patient no informed consent was obtained.

Of the remaining 30 patients, the median age was 70 (64 to 75) years and 27 patients (90%) were men. Six patients died and 24 survived until hospital discharge. Clinical characteristics of survivors and non-survivors are shown in Table 1. The non-survivors were significantly older, had a higher APACHE-II score and more sigmoid necrosis was observed. Blood-loss, lowest systolic blood pressure and suprarenal clamping did not significantly differ between survivors and non-survivors.

Table 2 displays the intra- and postoperative complications of the non-survivors. The six non-survivors died on days 2, 3, 4, 12, 21 and 30 after RAAA repair. In three of these patients the sigmoid colon had to be resected because of ischemic necrosis. Two patients had a culture proven infection. All patients died from multiple-organ failure.

**Figure 1:**
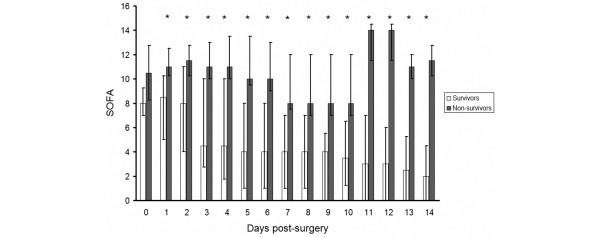
Sequential Organ Failure Assessment (SOFA) score after surgery for ruptured abdominal aortic aneurysm patients. The SOFA score was significantly (as indicated by asterisks) higher in non-survivors than survivors from day one post-surgery onwards.

The SOFA score did not differ significantly between survivors and non-survivors upon arrival in the ICU, but was significantly higher on days one through 14 in the non-survivors (Figure 1).

### C-reactive protein and white blood cell count

The median C-reactive protein level increased postoperatively. In non-survivors and survivors C-reactive protein (mg/dl) was 84 versus 31 on day 1, 283 versus 190 on day 3, 212 versus 157 on day 5 and 167 versus 159 on day 7. The median white blood cell count (× 10^9^/l) was 9.6 versus 10.0 on day 1, 7.8 versus 9.5 on day 3, 7.9 versus 9.4 on day 5 and 12.6 and 10.0 on day 7 in non-survivors and survivors, respectively. All differences were non-significant.

### Cytokine production

**Figure 2:**
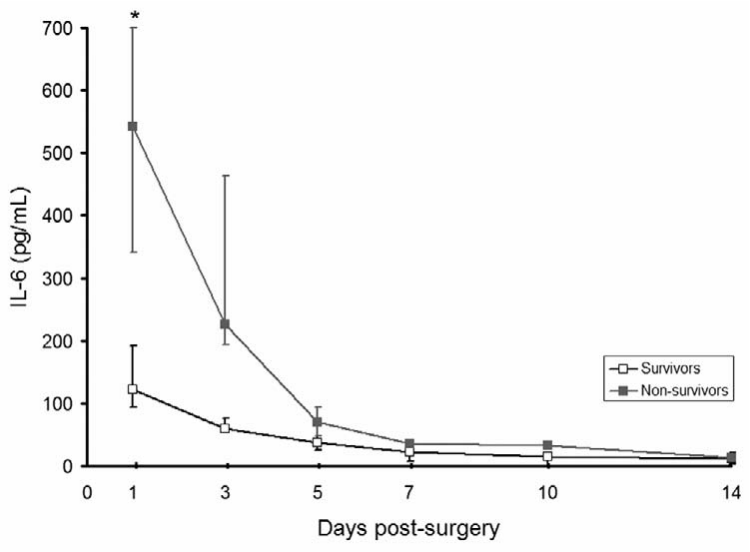
IL-6 after ruptured abdominal aortic aneurysm repair. Levels of IL-6 (normal value < 20 pg/ml) were significantly higher on day one in non-survivors. *p < 0.05.

**Figure 3:**
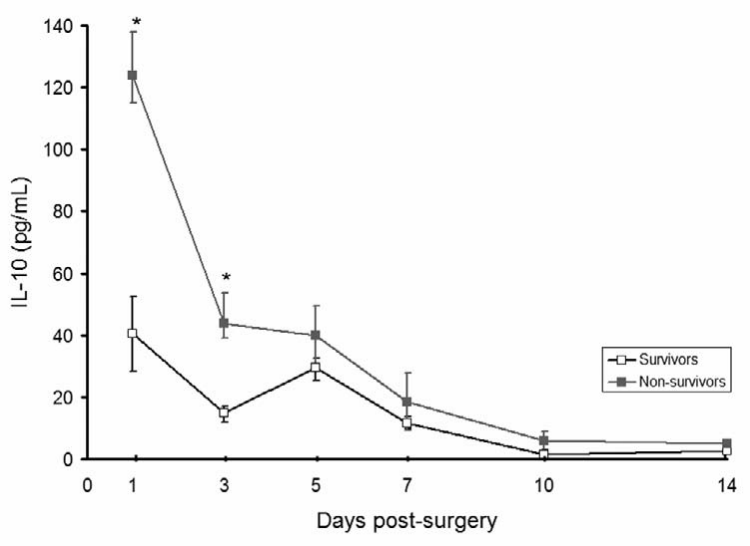
IL-10 after ruptured abdominal aortic aneurysm repair. Levels of IL-10 (normal value < 10 pg/ml) were significantly higher on days one and three in non-survivors. *p < 0.05.

IL-6 and IL-10 were elevated in all RAAA patients post-surgery (Figures 2 and 3). Median IL-6 was significantly higher in non-survivors versus survivors on day 1; median (interquartile range) 543 pg/ml (90 to 701) versus 122 pg/ml (39 to 137), p = 0.03. IL-10 was significantly higher on days 1 and 3 post-surgery in the non-survivors (p = 0.03 for both days).

### HLA-DR expression on monocytes

**Figure 4:**
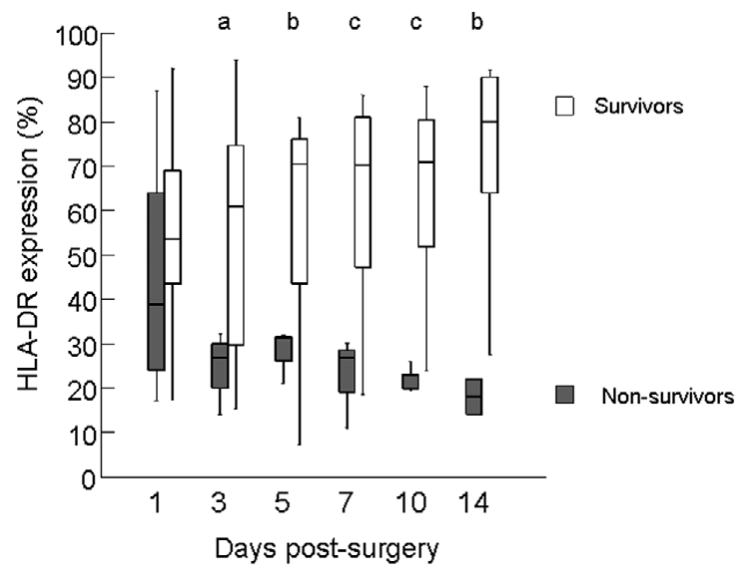
HLA-DR expression on monocytes after ruptured abdominal aortic aneurysm repair. The expression of HLA-DR on monocytes (normal range 76% to 96%) of patients after RAAA is sharply and significantly decreased from day three post-surgery onwards. ^a^*p *= 0.04; ^b^*p *= 0.02; ^c^*p *< 0.01.

On day one after surgery HLA-DR expression on monocytes was comparable in survivors and non-survivors, and significantly lower than the 76% to 96% observed in healthy controls. In survivors, HLA-DR expression rose to normal levels, whereas it decreased further and remained low in non-surviving patients. Percentages of monocytes expressing HLA-DR were significantly lower on days 3, 5, 7, 10 and 14 in the patients who died (Figure 4) compared to survivors. No significant differences were found between HLA-DR expression in the patients who developed infections (two non-survivors and seven survivors) and those who did not develop infections.

HLA-DR expression on days 1, 3, 5, 7, 10 and 14 had a significant negative correlation with the SOFA score on these subsequent days. After z-transformation, mean r = -0.416, 95% confidence interval (CI; -0.56 to -0.25), *p *< 0.01. The correlation coefficient between HLA-DR expression and IL-6 was r = -0.055, 95% CI (-0.26 to 0.15), *p *= 0.60. The correlation coefficient between HLA-DR expression and IL-10 was r = -0.078, 95% CI (-0.28 to 0.13), *p *= 0.47.

## Discussion

This study shows that, in the first days after RAAA repair, patients develop a generalised increase in immunoregulatory cytokines as reflected by elevated levels of IL-6 and IL-10. HLA-DR expression on monocytes is reduced and remains consistently low in non-survivors, while it returns to normal levels in survivors. Early high levels of IL-6 and IL-10 and subsequently reduced HLA-DR were all associated with multiple-organ failure and death.

Several studies have shown that a severe inflammatory response is associated with multiple organ failure and poor outcome in RAAA patients [9,17,18]. It is believed that, in RAAA patients, haemorrhagic shock, surgical trauma and ischemia reperfusion injury all contribute to this overwhelming inflammatory response. Blood-transfusions and surgery for sigmoid necrosis may also modulate the inflammatory response [19,20]. Our study confirms the presence of such an inflammatory response by demonstrating an increased production of IL-6. Furthermore, the SOFA score was significantly higher in the non-survivors from day 1 through day 14, with an increase in difference compared to survivors from day three (Figure 1). The anti-inflammatory cytokine IL-10 was significantly higher on days 1 and 3 in the non-survivors. Our findings are in accordance with a study described by Lekkou and colleagues [21] who studied 30 patients with severe sepsis and noted that HLA-DR expression was lower in non-survivors. Furthermore, these authors also found an initial high level of IL-6 in non-survivors and high IL-10 on days 3, 10 and 13. The association of an initial high level of IL-6 with organ failure and poor outcome is confirmed in patients with sepsis, after trauma and AAA patients [22-25]. The initial increase in IL-10 levels is also described in patients after orthopaedic trauma and pancreatitis [26,27]. The initial hyperinflammatory state followed by immunoparalysis, expressed as a prolonged increase in IL-10, could not be confirmed in these patient groups. In patients with septic shock, Monneret and colleagues described a significantly lower HLA-DR expression and higher IL-10 in non-survivors [28]. Caille and colleagues [29] described that HLA-DR expression was low in septic shock but not decreased in patients with haemorrhagic shock. One might conclude that our data from RAAA patients are in contrast with these findings. However, Caille and colleagues described patients with trauma and postpartum haemorrhage; these patients do not suffer from an additional ischemia reperfusion injury. RAAA patients experience haemorrhagic shock and ischemia reperfusion injury simultaneously.

The causal relationship between low HLA-DR expression and poor outcome in ICU patients remains an interesting point of discussion. In patients with sepsis, low HLA-DR expression is associated with monocyte deactivation, an anti-inflammatory cytokine profile, infectious complications and death [30,31]. In patients with RAAA we could only partially confirm these findings. As shown in Figure 4, a decrease in HLA-DR expression on monocytes is associated with a poor outcome. However, the presence of a sustained anti-inflammatory response in these patients is difficult to envisage, considering the fact that IL-10 levels are low from day three post-surgery, following an initial rise. In our series, only two non-survivors developed culture-proven infection. One of these patients had necrosis of the sigmoid colon and the other developed a wound infection in the presence of extensive organ failure. The majority of the patients died from multiple organ failure, not from overwhelming infection due to functional immunosuppression (Table 2). In theory, early death from multiple organ failure may have prevented the onset of severe infection, but this remains speculative. Low HLA-DR expression in the non-survivors might also be the result of their older age, although this correlation could not be confirmed [32,33]. Alternatively, low HLA-DR expression may not be causally related to mortality. As such, HLA-DR expression may be no more than a coincidental finding. Blood-loss and sigmoid resection may also significantly alter the immune response and HLA-DR expression, although no significant differences were found between these factors in survivors versus non-survivors.

What can we do to improve the survival of RAAA and other surgical patients? Since blood-loss and duration of surgery are related to the development of multiple organ failure, meticulous technique will increase the chances of survival. The recent advances in endovascular surgery are promising [34-36], although long-term durability is unknown [37-40]. Because of the shortcomings of endovascular surgery, RAAA patients with a severe inflammatory response after open surgery will continue to be presented to the ICU and a significant number of them will not survive. In theory, hydrocortisone might also lead to a better outcome in RAAA patients. In patients with sepsis and relative adrenal insufficiency, hydrocortisone suppletion improves prognosis [41]. In addition Keh and colleagues [42] showed that hydrocortisone restored haemodynamic stability and modulated the immunological response. These effects might also benefit RAAA patients since adrenal insufficiency can be identified in a significant amount of them [43]. The clinical effect of hydrocortisone suppletion in RAAA patients needs to be evaluated further.

Our study has its limitations, mainly because of the small patient numbers and the relatively low percentage of non-survivors. On the other hand, these low numbers were enough to reach statistical significance on HLA-DR expression. This is probably related to the fact that RAAA patients present as an homogenous group with a well-defined insult, as reflected in similar values for blood-loss, lowest systolic blood pressure and suprarenal clamping.

## Conclusion

Patients with RAAA displayed a severe inflammatory response post-surgery, with markedly increased immunoregulatory cytokines. HLA-DR expression was low in non-survivors from the day of surgery onwards. In contrast to survivors, in whom levels returned to normal values, it remained low. Organ failure was present in non-survivors from day 1 and was the primary cause of death. A relationship between impaired monocyte function and death from infectious causes was not apparent in our series. More likely, the severe initial insult in non-survivors is probably responsible for both their low HLA-DR expression on monocytes and their onset of fatal multiple organ failure.

## Key messages

• RAAA patients all demonstrate a generalised increase in immunoregulatory cytokines in the first days after surgery, displayed by increased production of IL-6 and IL-10.

• HLA-DR expression on monocytes decreased after surgery, but recovered to normal levels in survivors.

• Low HLA-DR expression and high IL-6 and IL-10 levels in the first days after surgery were associated with multiple organ failure and death.

• We found no evidence for low HLA-DR expression on monocytes and death from secondary infections.

## Abbreviations

APACHE = Acute Physiology and Chronic Health Evaluation; CI = confidence interval; ELISA = enzyme-linked immunosorbent assay; ICU = intensive care unit; IL = interleukin; RAAA = ruptured abdominal aortic aneurysms; SOFA = Sequential Organ Failure Assessment.

## Competing interests

The authors declare that they have no competing interests.

## Authors' contributions

JWH participated in data collection and statistics of the studied patients, did most of the writing of the article, and coordinated the study. APvdB contributed to the format of the study and writing of the article and assisted and participated in data collection and statistics. ELGV contributed to the format of the study and writing of the article. MWNN assisted and participated in data collection and statistics and contributed to writing of the article. JJAMvdD contributed to the format of the article and assisted in data collection. THT contributed to the design of the study and data collection. JHZ contributed to the data collection and statistics of the studied patients and writing of the article and coordination of the study. All authors gave final approval of the version to be published.

## Figures and Tables

**Table 1:** Characteristics of the survivors and non-survivors

	Survivors (*N *= 24)	Non-survivors (*N *= 6)	*P*
Demographic characteristics			
Age	68 (64–72)	78 (73–79)	<0.01
Sex (male)	21 (88%)	6 (100%)	NS
Intra-operative data			
Lowest systolic blood pressure (mmHg)	77 (53–90)	65 (23–79)	NS
Blood-loss (litres)	3.8 (1.6–5.4)	4.8 (2.0–12.9)	NS
Suprarenal clamping (yes)	5 (21%)	2 (33%)	NS
Post-operative data			
APACHE-II score	13 (9–16)	19 (16–23)	0.01
Re-operations (total)	10	4	NS
Sigmoid resection	2 (8%)	3 (50%)	0.04
Rebleeding	0	0	NS
Lower leg amputation	1 (4%)	0	NS
Infectious complications (total patients)	7	2	NS
Septic shock	(17%)	2 (33%)	NS
Abdominal infection	3 (13%)	1 (4%)	NS
Pneumonia	4 (17%)	0	NS
CVC infection	2 (8%)	0	NS
Wound infection	0	1 (4%)	NS
Renal replacement therapy	4 (17%)^a^	2 (33%)^b^	NS
Hydrocortisone treatment^c^	3 (13%)	1 (17%)	NS
ICU length of stay (days)	7 (2–16)	8 (3–23)	NS
Hospital length of stay (days)	20 (11–33)	8 (3–23)	NS

Data are given as medians with interquartile range, or absolute number of patients with percentage of the total population. ^a^All patients had a full recovery of renal function at hospital discharge. ^b^In an additional two patients renal replacement therapy was indicated but not performed because it was considered futile. ^c^Hydrocortisone treatment was initiated for relative adrenal insufficiency. APACHE, Acute Physiology and Chronic Health Evaluation; CVC infection, central venous catheter related infection; ICU, intensive care unit; NS, non-significant.

**Table 2:** Clinical data of the six non-survivors

Patient	Age (years)	Died on day	Resuscitation during surgery	Blood-loss (l)	Complications	Organ failure	Infectious complication
1	79	2	-	7.0		Respiratory and renal	-
2	80	3	-	9.5	Sigmoid necrosis, ischemic lower legs	Respiratory, cardiovascular and renal	-
3	74	4	-	2.0		Respiratory, cardiovascular and renal	-
4	79	12	-	2.5	Sigmoid necrosis	Respiratory, hepatic, cardiovascular and renal	Enterococci in blood culture
5	79	21	-	3.0	Wound infection	Respiratory, cardiovascular and renal	Proteus mirabilis in wound and blood
6	76	30	+	25.0	Sigmoid necrosis	Respiratory, cardiovascular and renal	-
